# Beneficial Effect of Foot Plantar Stimulation in Gait Parameters in Individuals with Parkinson’s Disease

**DOI:** 10.3390/brainsci10020069

**Published:** 2020-01-27

**Authors:** Lorenzo Brognara, Emmanuel Navarro-Flores, Lorenzo Iachemet, Nuria Serra-Catalá, Omar Cauli

**Affiliations:** 1 Department of Biomedical and Neuromotor Science, University of Bologna, Via Ugo Foscolo 7, 40123 Bologna, Italy; lorenzo.brognara2@unibo.it (L.B.); lorenzoiachemet93@gmail.com (L.I.); 2 Frailty and cognitive impairment organized group, University of Valencia, 46010 Valencia, Spain; emmanuel.navarro@uv.es; 3 Department of Nursing, University of Valencia, c/Jaume Roig s/n, 4610 Valencia, Spain; 4 Geroresidencais, La Saleta, Armonea Group, 46015 Valencia, Spain; ocauli@yahoo.com

**Keywords:** asymmetry, stride length, foot, rehabilitation, medical device, 3D printing, additive manufacturing

## Abstract

New treatments based on peripheral stimulation of the sensory-motor system have shown to be promising in rehabilitation strategies for patients with neurological disorders, including Parkinson’s disease (PD), especially in regards to reducing gait impairment, and hence, the incidence of falls. The aim of this study was to evaluate the change in several gait parameters measured by sensor inertial measurement in PD patients after acute plantar stimulation, under the distal phalanx of the big toe, and underneath the head of the first metatarsal joint of both feet, using a 3D printing insole. In order to assess whether the effects are selective for PD patients, we compared the effect of the treatment in a control group (age-matched) consisting of patients with other neurological disorders which also displayed gait and balance impairment, and a similar cognitive function, depressive symptoms, body mass index, and comorbidity burden observed in the PD group. Plantar foot stimulation in PD patients eliminated the significant (*p* < 0.05) alterations existing in stride asymmetry and in stride variability. When comparing the effects of post-plantar stimulation with the respective basal level, considered as 100% in both groups, we observed a significant (*p* = 0.019, Mann–Whitney test) increase in stride length compared to basal in the PD group and control group. No significant effects of foot plantar stimulation were observed in any of the gait parameters in the control group. Plantar foot stimulation has a positive effect on the step and stride length, and has a positive effect on walking stability, measured by the increase in stride length. No significant effect was observed on bradykinesia because it did not improve walking velocity. These findings indicate that foot plantar stimulation using a 3D printing insole seems to generate a more stable walking pattern in PD patients, with an interesting applicability, and a low-cost, for reducing gait impairment in PD patients.

## 1. Introduction

Parkinson’s disease (PD) is a progressive and complex neurological disorder characterized by neural degeneration of the substantia nigra pars compacta, which results in a decreased dopamine release in basal ganglia [1]. PD affects approximately 0.3% of the population; disease prevalence increases to 1% of subjects who are 65 and older. Traditional management focuses mainly on drug therapy by administrating the dopamine precursor L-dopa and dopamine receptor agonists; thus, reducing symptomatology [2]. However, over time chronic levodopa treatment can be associated with decreased response and motor complications such as “wearing-off phenomena” and dyskinesia [1,2]. Postural and motor impairments are among the most debilitating symptoms for patients with PD, directly affecting their quality of life [3]. Postural instability can be defined as the impairment of balance that compromises the ability to maintain or change posture, being considered one of the main features of Parkinson’s disease, along with resting tremors, rigidity, and bradykinesia [4]. The effects of L-dopa on postural instability and balance control remain successful for a limited period of time [5,6]. Shaafsma et al. [7] studied the relationship between levodopa therapy and falls in PD patients, reporting a significantly reduced stride time variability, which has been found to increase the fall incidence in individuals with PD. Decreased stride length is considered the most prominent feature, and is often accompanied by reduced walking speed, and a longer duration of the double-support phase [8,9,10]. Further, Blin et al. report that stride length variability seems to show a correlation with the clinical stage of disease, and results significantly higher in PD patients compared to a control group [7]. In recent years, new rehabilitation strategies have been proposed in order to provide alternative approaches for gait and postural impairments. Previous studies have demonstrated the correlation between sole sensitivity deficit and impaired postural control in PD patients, proving the key role of peripheral alterations of the sensory-motor system in balance and motor symptoms [11,12]. Various types of sensory receptors, such as proprioceptors, give continuous feedback to the central nervous system. Various studies, with different methods, have been investigating the effects of peripheral bottom-up stimulation in order to reduce motor impairment in PD patients [13,14,15,16,17,18]. By enhancing the sensory feedbacks coming from the feet, bottom-up stimulation allows patients to better integrate peripheral inputs with motor control response during gait, producing a stimulating effect of central nervous system areas involved in human movement [18]. Recent studies have shown the effectiveness of plantar mechanical stimulation in PD patients by applying punctual pressure in two specific areas of each foot (bottom-up rehabilitation) [19,20,21,22]. Furthermore, a pilot study by Quattrocchi and co-workers demonstrated the effectiveness of a device for the motor rehabilitation in PD, based on non-invasive stimulations given via controlled mechanical impulses in specific areas of both feet [18]. The application of this device increased resting state functional connectivity of the sensory motor cortex, nucleus striatum, and cerebellum in PD patients treated with automated mechanical peripheral stimulation (AMPS) [18]. These results suggest that AMPS increases brain functional connectivity by facilitating brain compensatory pathways useful to manage PD motor symptoms. Although literature shows that AMPS is an effective strategy for treating gait impairments in these patients, the high costs of the device represent a great limit for usability, being used mainly in private facilities rather than being implemented in the public healthcare system. Based on these findings, the aim of our study is to assess the effect of mechanical peripheral stimulation (MPS) by means of customized 3D printed insoles, and to verify if it promotes changes in several spatiotemporal parameters of the gait. In this study, we evaluated gait impairments by assessing different spatiotemporal gait parameters, some of which have not been investigated in previous studies, such as stride variability and asymmetry. Such variables are useful to assess walking stability and rhythmicity, and have been correlated to fall risk [23,24]. In addition, we wish to analyze if the effects were different when comparing the results in the PD group with a control group formed by individuals with other neurological disorders.

## 2. Material and Methods

A cross-sectional pilot study was performed in individuals with a residential profile, who were institutionalized in long-stay centers for the elderly in the province of Valencia (GeroResidencias La Saleta, Valencia, Spain). The study sample was recruited between October 2018 and June 2019. In accordance with the requirements established by the declaration of Helsinki, written consent was obtained from each person and their relatives, after having been informed in a clear and simple way about the purpose of the study and the procedures involved. The study protocol was approved by the Human Research Ethics Committee at the University of Valencia (Reference: H38417528).

Inclusion criteria were subjects of both sexes who fulfilled the following criteria: (1) age of 65 years or older; (2) subjects able to walk autonomously or with the assistance of a cane or a walker; (3) antiparkinsonian treatment at a stable and optimized daily dosage during the three months prior to the study; (4) absence of dopaminergic long-lasting residual effects. Exclusion criteria were: (1) dementia established on the basis of the Mini-Mental State Examination; (2) history or presence of peripheral sensory neuropathy; (3) any peripheral musculoskeletal conditions that may alter balance and/or gait; (4) lower limb injuries in the previous 6 months; (5) history of neurosurgery or orthopedic surgery; (6) recent (<3 months) hospitalization, diagnosis of cancer, blindness.

Each individual underwent a complete clinical, geriatric, and functional assessment. Four validated scales were used to evaluate the functional and cognitive areas: the Barthel Index, Tinetti scale, Yesavage scale, and a mini-mental test (MEC) [25,26,27,28,29]. The Barthel Index assesses the ability to perform the activities of daily life (ADLs), and measures independence with 10 items, with a score range of 0–100. The items assessed are: feeding, bathing, grooming, dressing, urine and fecal continence, toilet use, transfers (bed to chair and back), mobility (on level surfaces), and ability to use stairs. A lower score indicates greater dependence, while a higher score indicates greater independence, with 0 representing total dependence and 100 total independence. The Tinetti assessment tool is an easily administered task-oriented test that measures an older adult’s gait and balance abilities. A three-point ordinal scale, ranging from 0–2. The “0” indicates the highest level of impairment and the “2” indicates the individuals’ independence (Total Balance Score = 16; Total Gait Score = 12; Total Test Score = 28). The interpretation of the Tinetti score is the following: score 25–28 = low fall risk; score 19–24 = medium fall risk, and a score <19 = high fall risk. The Yesavage scale evaluates the depressive symptoms present in the elderly. We used the reduced version, composed of 15 dichotomous response (yes or no) items, with scores ranging from 0 to 15, where a score of over 5 indicates the probable presence of depression. The presence of depression was also dichotomized by reviewing the medical records for a clinical diagnosis of depression (including antidepressant and other psychotropic drug treatments). The MEC is the Spanish version of the Mini-Mental State Examination (MMSE), and comprises of 11 items that screen cognitive impairment by assessing five cognitive areas: orientation (temporal and spatial), attention and calculation, word recall, language, and visuospatial abilities. The maximum MEC score is 35 points, and scores lower than 30 points suggest the presence of cognitive impairment. Specifically, normal cognitive function scores 30–35 points, borderline cognitive deficits score 25–29 points, mild cognitive impairment scores 20–24 points, moderate cognitive impairment scores 15–19 points, and severe cognitive impairment receive ≤ 14 points [24,25,26,27]. The Charlson Comorbidity Index adjusted for age was calculated with a free online tool (https://www.mdcalc.com/charlson-comorbidity-index-cci). The PD group was assessed using the Unified Parkinson’s disease Rating Scale (UPDRS III). UPDRS is a rating scale that allows one to follow the severity and progression of the disease. Section III refers to clinically scored motor evaluation. It has ratings ranging from 0 to 4, in which the severity of the symptoms is rated 0 (normal) to 4 (severe). The following subdomains of the UPDRS scale were analyzed: mentation, behavior, mood (Mental-UPDRS), and complications of therapy (Therapy-UPDRS). PD patients were evaluated in OFF state (12 h without antiparkinsonian treatment).

The treatment consisted of staying in the orthostatic position (stable standing), wearing insoles, which present the stimulations, for 5 min. While realizing this procedure, the podiatrist checked the activation of the monosynaptic reflex in the tibialis anterior muscle, as described in previous studies. Every patient (PD group and Control group) wore the custom insoles, while clinical evaluations and gait analyses were performed before and immediately after the treatment (time elapsed between pre- and post-test was approximately 30 min). Subjects were instructed to walk at their preferred speed before and after the plantar stimulation. Gait analysis was carried out by means of a 7-Meter Walk Test (7MWT) while wearing portable inertial sensors. Gait was assessed using mGait, a system made of two inertial measurement units worn on the shoes (attached to shoelaces with a Velcro strap), and connected via Bluetooth to a smartphone app. This system is part of the mTest^3^ product (mHealth Technologies, Bologna, Italy). Data were collected with an inertial sensor measurement system consisting of two sensor units, by using wearable inertial sensors (IMUs), which allowed to obtain several spatial-temporal parameters, and estimate, with great accuracy, the kinematic parameters as well as the position, the acceleration, and the speed produced by the movement. The device is composed of a transducer, known as a micro-electro-mechanical system (MEMS) that detects movement and transforms the mechanical signal into an electric one using an algorithm. This tool can be used to determine acceleration and, via signal integration, speed and displacements. The sensors are composed of a tri-axial accelerometer, tri-axial gyroscope, and magnetometer that can provide quantitative and meaningful data about patient performance that can be useful to improve clinical interpretation, and to evaluate treatments in a more objective way [30]. Subjects were instructed to walk at their normal gait-speed for 7 m while wearing the inertial sensors. This procedure took place two times, before and immediately after MPS, which was delivered by 3D-printed customized insoles. All patients were asked to walk at a self-selected walking speed for 7 m; the following spatiotemporal parameters were obtained: stride variability, asymmetry, gait speed, stride length, stride duration, cadence, swing phase, single support and double support phase, and pitch contact. The descriptions of these variables are shown in Table 1.

**Figure 1:**
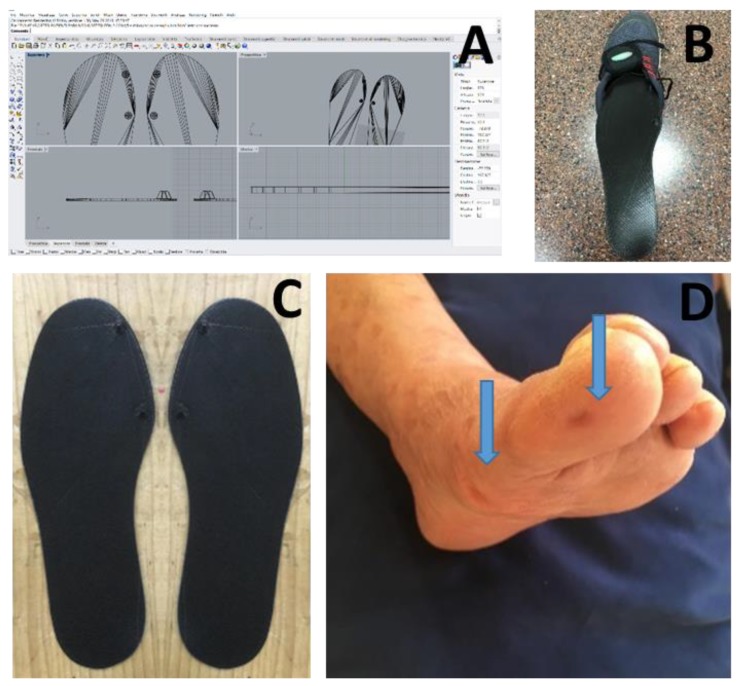
Details of the realization process of the customized 3D printed insoles design used in this study, (**A**) and the insoles (**C**) inside the shoe with the inertial measurement unit attached to shoelaces with a Velcro strap (**B**). Blue arrows indicate the effect of blunted cones on the foot after mechanical peripheral stimulation on the plantar surface of the first metatarsal joint and distal phalanx (**D**).

The podiatrist, after having assessed the clinical general conditions of the patient’s foot, scanned the patient’s feet using a 3D Sense laser scanner (3D Systems, Rock Hill, South Carolina, USA) in order to obtain 3D geometrical data of the subject’s foot. The scans were uploaded into 3D CAD software (Rhinoceros; McNeel, Seattle, WA, USA), which was useful to create and generate the 3D model of the custom insole with the mechanical stimulation (two blunted cones located under the distal phalanx of the big toe and underneath the head of the first metatarsal joint of both feet). The customized insoles were manufactured by means of a Delta Wasp 4070 3D printer (CSP s.r.l. Massa Lombarda, Italy), based on Fused Deposition Modelling (FDM). We chose this technology in order to standardize insoles and to guarantee higher reproducibility when compared with a traditional, handmade process (Figure 1).

**Figure 2:**
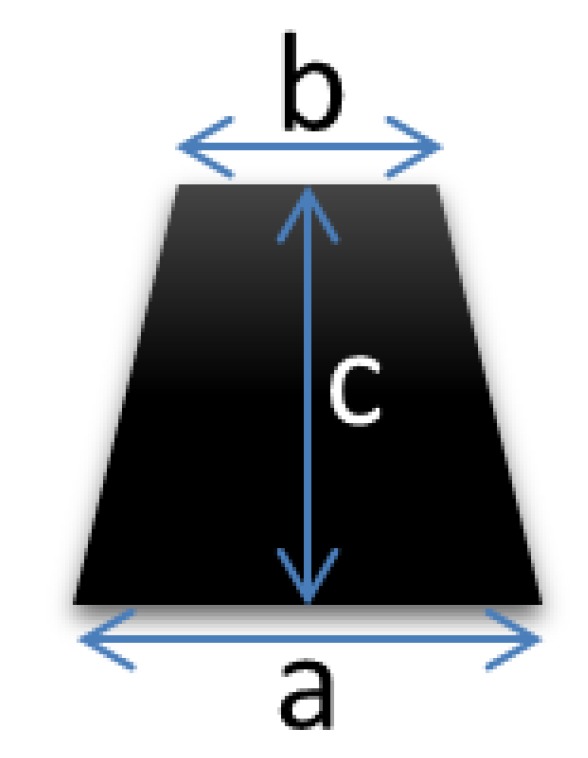
Blunted cone’s size (a: 5 mm, b: 2 mm, c: 7 mm).

Previous studies investigated the responsiveness of mechanical peripheral stimulation on specific areas of the foot (first metatarsal head, plantar surface of big toe); thus, reducing motor symptoms by stimulating the somatosensory system [19,20,21,22,31]. In such studies, results were compared with age-matched control groups in order to identify differences in gait pattern between PD patients and healthy subjects. The aim of the study was to stimulate foot plantar through the application of customized 3D-printed insoles that present two blunted cones (Figure 2) in similar plantar area feet analyzed in other reports [31]. This ensures usability, preserves adherence, and avoids discomfort or stigma.

The quantitative variables were subjected to a descriptive analysis using mean and standard deviation. Likewise, a descriptive analysis was also performed for the qualitative variables, based on frequency distributions. The Kolmogorov–Smirnov test revealed non-normal data distribution for quantitative variables; therefore, we used nonparametric tests. The differences between the means of PD and control groups were analyzed using non-parametric tests (Mann–Whitney test). The analysis of changes between parameters before and after plantar stimulation in the same subject were evaluated with the Wilcoxon rank test. The confidence level used for all analysis was 95%, with a statistical significance of *p* < 0.05. The IBM SPSS statistical package (version 25.0) was used for all statistical analysis.

## 3. Results

### 3.1. Characteristics of the Sample

The study selected 12 patients affected by idiopathic Parkinson’s disease (6 female, 6 males). The average characteristics of the PD group were the following: age = 79.8 ± 7.7 years old; BMI = 26.4 ± 4.4 kg/m^2^; total UPDRS = 51.9 ± 16.4; and 12 age-matched control groups (8 females, 4 males), with a total UPDRS = 14.3 ± 10.8; age = 82.9 ± 8.5 years old; BMI = 27.0 ± 7.0 kg/m^2^. The control group consisted of eight patients with post-stroke motor deficits as long-term neurological sequelae, two patients with vascular Parkinsonism, one patient with motor deficits after post-herpetic encephalitis, and one patient with normotensive hydrocephalia. There was no significant difference between the two groups in BMI, ADL activity measured with performance in the activities of daily life, mini-mental examination test, Charlson Comorbidity Index (age-adjusted), and depressive symptoms. However, the PD group displayed significant (*p* = 0.01) lower scores in gait and balance compared to the control group (Table 2).

### 3.2. Differences in Gait Parameters between PD Patients and Control

**Figure 3:**
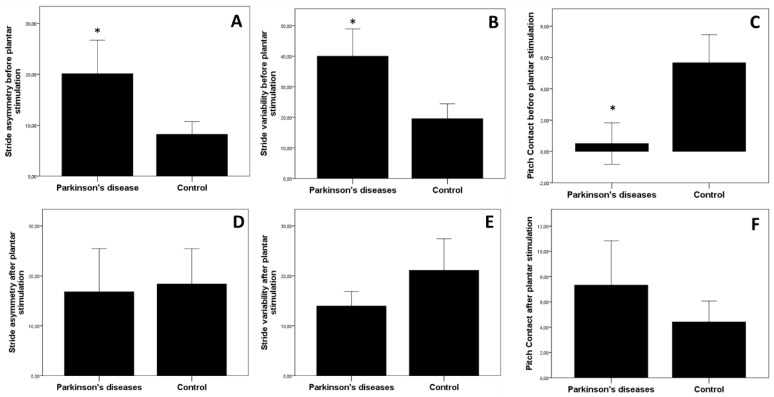
Comparison between spatiotemporal parameters of gait before and after mechanical peripheral plantar stimulation. Panels (**A**–**C**) represent the mean values before, and panels (**D**–**F**) after plantar stimulation. Plantar stimulation was maintained in stand position for 5 min. * *p* < 0.05 Mann--Whitney test.

In the pre-test, there was a significant difference in gait parameters between the PD group and control group for asymmetry: (PD. 20.1 ± 22.8 s; Control: 8.2 ± 8.7 s, *p* = 0.030, Mann–Whitney test) (Figure 3A), for variability (PD: 40.1 ± 30.8%; Control: 19.6 ± 16.8%, *p* = 0.033, Mann–Whitney test) (Figure 3B), and Pitch contact (PD: 0.5 ± 4.6°; Control: 5.7 ± 6.2, *p* = 0.040, Mann–Whitney test) (Figure 3C).

No significant differences were found between the PD and control groups for the other gait parameters (*p* > 0.05 in all cases). In the PD group, the severity of asymmetry was significantly and directly correlated with total-UPDRS score (rho = 0.549; *p* = 0.031), and the subdomain ADL-UPDRS (rho = 0.542; *p* = 0.024), but not with Mental-UPDRS (rho = −0.231; *p* = 0.469), or Motor-UPDRS (rho = 0.497; *p* = 0.101). The severity of variability was significantly and directly correlated with the total UPDRS score (rho = 0.606; *p* = 0.031), but not with the Mental-UPDRS (rho = 0.359; *p* = 0.133), the subdomain ADL-UPDRS (rho = 0.286; *p* = 0.386), or with Motor-UPDRS (rho = 0.107; *p* = 0.612). The severity of pitch contact was not significantly correlated with the total-UPDRS score (rho = 0.049; *p* = 0.879), or with the subdomain ADL-UPDRS (rho = −0.047; *p* = 0.885), Motor-UPDRS (rho = 0.236; *p* = 0.461), or with Mental-UPDRS (rho = −0.122; *p* = 0.706). No significant correlation was found for other clinical variables, such as comorbidity index, BMI, or gender (*p* > 0.05 in all cases).

### 3.3. Effect of Foot Plantar Stimulation in Gait Parameters

**Figure 4:**
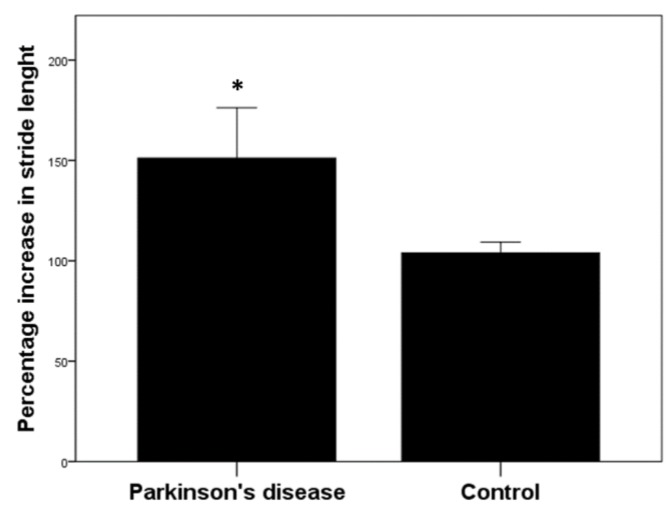
Comparison of percentage increase in stride length between two groups. Percentage change in stride length of plantar stimulation respect to basal (before plantar stimulation) level. * *p* < 0.05 Mann–Whitney test.

When considering the two groups (PD and Control group) pooled together, foot stimulation did not significantly change any gait parameter when comparing the value after plantar stimulation (expressed as a percentage change of basal value before plantar stimulation) (*p* > 0.05, Wilcoxon rank test). When analyzing gait parameters pre- and post-foot stimulation in Control group, no significant differences were observed (cadence, *p* = 0.825; stride length, *p* = 0.672; gait speed, *p* = 0.885; stride duration, *p* = 0.382; pitch contact, *p* = 0.110; asymmetry, *p* = 0.088; swing phase, *p* = 0.661; variability, *p* = 0.710, Wilcoxon rank test). In contrast, in the PD group, plantar stimulation eliminated the significant difference existing in stride asymmetry (*p* = 0.478, Mann–Whitney test) (Figure 3D), in stride variability (*p* = 0.551, Mann–Whitney test) (Figure 3E), and in pitch contact (*p* = 0.630, Mann–Whitney test) (Figure 3F), between PD and the control group. When comparing the effects of post-plantar stimulation, with the respective basal level considered as 100% in both groups, we observed a significant (*p* = 0.013, Mann–Whitney test) increase in stride length compared to basal in the PD group and control group (PD: 130.7 ± 41.1; Control: 103.9 ± 18.6) (Figure 4). Whereas the percentage change over basal levels for the other gait parameters did not change significantly between the two groups (data not shown). 

## 4. Discussion

Non-pharmacological treatment, such as physiotherapy and rehabilitation, are fundamental to tackle motor symptoms together with dopamine-mimetic therapy, in particular for symptoms less responsive to pharmacological treatment, such as gait impairment, walking disturbances, and impaired balance, since these symptoms tend not to respond adequately to traditional treatments. It has been previously reported that individuals with PD have signs of sensory impairment, both peripherally and centrally, and sensory processing deficits contribute to movement disorders in subjects with PD [11,32,33]. Based on these findings, different methods of plantar sensory stimulation have been investigated to improve movement alterations in PD, with promising results for clinical applications [14,34,35,36]. The Pagnussat and coworkers procedure used a stimulation based on the application of a pressure via rounded tips in the four target areas of the feet (two in each foot, corresponding to the head of the big toe and the base of the first metatarsal bone). Interestingly, the stimulation pressure to obtain such benefit was in the range of 0.3/0.9 N/mm^2^, similar to the pressure stimulation applied in our study. Jenkins et al. [14] compared the effects of a facilitatory (ribbed) insole and a conventional (flat) insole while walking 20 feet in PD patients. The facilitatory insole produced a significant increase in single-limb support time by normalizing the muscle activation sequence of the tibialis anterior at the time of initial ground contact. Lirani-Silva et al. [35] observed benefits offered by textured insoles on plantar sensation and stride length in people with PD after continuous use. A similar improvement in stride length was observed in PD compared to the control group in our study, confirming that plantar stimulation may improve postural instability seen in PD [14,36]. Recent studies have shown that punctual pressure in two specific areas of each foot (bottom-up rehabilitation) is sufficient to improve gait and stability in PD patients [19,20,21,22]. 

Findings by Kleiner et al. show that after AMPS, patients performed the Timed Up and Go tests faster when compared to pre-treatment evaluation, while also improving their walking stability; thus, reducing the risk of falling. These changes may lead to an overall improvement in gait pattern and stability, suggesting that the use of these types of facilitatory insoles may be a useful treatment strategy for improving gait quality in individuals with Parkinson’s disease. Increasing plantar cutaneous sensitivity will improve spatiotemporal parameters and muscle contraction patterns of gait among individuals with PD. Our protocol of foot plantar stimulation agree with the results obtained by other reports using different protocol of plantar stimulation [19,20,21,31]. Even in our experimental protocol, the pressure of the plantar stimulation could depend on the weight of the subjects. We did not find any statistical association between the weight or the height of the patients, and the improvement in stride length. In this study, patients were assessed using mGait (mHealth Technologies, Bologna, Italy), a system for gait analysis based on wearable sensors, providing a large number of spatiotemporal gait variables, some of which are not discussed in the previous studies, such as variability and asymmetry. Such variables are useful to assess the walking stability and rhythmicity correlated to fall risk [23,24]. While most of the spatiotemporal parameters are not disease-specific, and tend to be age-related, previous studies have shown that the variability of stride length is increased in patients with PD, but not in age-matched healthy subjects [8,37]. Also, Blin et al. have shown a significant correlation between stride length variability and clinical stage of the disease [7]. Since no other studies have investigated such parameter, before and after mechanical peripheral stimulation, it could represent a disease-specific value; thus, giving new insights in the understanding of PD gait patterns. Asymmetry is another parameter we took into account that has not been investigated in previous studies. It could represent an indicator of gait rhythmicity, useful to detect discrepancies between the patterns of both limbs during gait. This is the first study comparing variability of stride length before and after mechanical peripheral stimulation in PD patients. No previous studies have taken into account asymmetry as an indicator of walking rhythmicity. It can be useful to evaluate whether one side of the body is more affected by assessing discrepancies between gait patterns of each limb during walking. Blin et al. [8] also found that in patients with PD, variability of stride length increases as a function of the Hoehn and Yahr stage, which is closely related to balance disorders; thus, representing the parameter that best correlates with the clinical stage of disease. Pitch contact value represents the angle between the ground and the foot during heel contact. Patients with PD, by an inadequate heel clearance, lead a reduction of the pitch contact and may increase fall risk [38]. Although no previous studies mentioned such parameter, we took it into account as it seems to be a useful indicator of anterior tibialis contraction power and ankle range of motion during gait. Ankle dorsiflexion is a key element while initiating gait, as it allows the foot to strike on the ground in a position that facilitates sagittal plane motion during heel rocker; thus, preserving progressional momentum. A randomized-controlled trial by Sale and co-workers [39] using robot assisted gait training found similar improvements in gait parameters in PD patients, in particular for the beneficial effect on stride length. In contrast, our study did not find any significant effects on step length and velocity, which should be taken into account for future rehabilitative strategies in PD, in order to balance clinical benefits and the relative costs of these innovative techniques.

One limitation of the present study concerns the evaluation of acute effects of plantar stimulation on gait performance, but the duration of the effect and/or the presence of any effect saturation of the plantar stimulation over time might reduce the clinical impact of this rehabilitative approach. For these reasons, further studies should evaluate the neurological effect of chronic treatment. We are aware of the limited size of the study as it is the main limitation of our study, although, in our opinion, it was sufficient for a pilot study in order to create future research directions in this interesting field. Previous studies assessing the responsiveness of peripheral foot stimulation recruited a similar sample size as in the present study [18]. Pilot studies are important in order to avoid significant errors before implementing large scale studies; they aim to access feasibility, and to obtain preliminary data that can be used to design a relevant, economical, and statistically adequate large scale study [40,41]. However, the fact that the design, content, and results from most pilot studies remain unpublished is, in our opinion, unfortunate for two reasons: (1) The feasibility of the published data may prevent other researchers from making similar methodological mistakes, and thus wasting limited research resources; (2) Making pilot studies results available may allow others to avoid having to assess the feasibility of particular aspects of their proposed studies. The innovative 3D printing insoles are significantly less expensive for PD patients and healthcare providers compared with automated mechanical peripheral stimulation therapies recently reported in literature, by means of a commercial medical device [19,20,21,31]. It represents a new, low-cost and safe intervention that could be easily integrated in the current treatment protocol for PD patients.

## Figures and Tables

**Table 1:** The table shows all spatiotemporal parameters of gait analyzed during the pre- and post- mechanical peripheral stimulation.

Type of Recorded Variable (Unit)	Measurement
Variability (s)	Standard deviation of stride duration.
Asymmetry (%)	Speed difference between right and left side.
Gait speed (m/s)	Ratio between the length and duration of the stride.
Stride length (m)	Distance between successive ground contacts of the same foot.
Cadence (step/min)	Number of steps per minute (stride frequency).
Stride duration (s)	Time elapsed between the first contact of two consecutive footsteps of the same foot.
Swing phase (%)	The swing phase is that part of the gait cycle during which the reference foot is not in contact with the ground and swings in the air. It constitutes about 40% of gait cycle.
Single support and Double support	Double support (2 times 10%); when only one is in the support phase. Single support (40%), the second then being in oscillating phase.
Pitch Contact (°)	Contact angle between the foot and the ground during the first step contact.

**Table 2:** Psychogeriatric evaluation of study participants. We reported mean ± standard deviation. For comparisons between groups the U Mann–Whitney test. T was used.

	Parkinson’s Disease	Control	*P* Value
Performance in the activities of daily life (Barthel score)	57.9 ± 6.2	62.5 ± 3.2	0.23
Gait and balance (Tinetti scale)	12.7 ± 2.6	16.3 ± 1.1	0.01
Depressive symptoms (Yesavage scale)	3.7 ± 1.2	3.5 ± 0.6	0.65
Cognitive function (MEC score)	28.1 ± 2.6	29.6 ± 0.9	0.21
Charlson comorbidity (age-adjusted) index	5.8 ± 1.6	5.8 ± 0.5	0.71
